# Vascular responses of penetrating vessels during cortical spreading depolarization with ultrasound dynamic ultrafast Doppler imaging

**DOI:** 10.3389/fnins.2022.1015843

**Published:** 2022-11-16

**Authors:** Bao-Yu Hsieh, Yu-Chieh Jill Kao, Ning Zhou, Yi-Pei Lin, Yu-Ying Mei, Sung-Yu Chu, Dong-Chuan Wu

**Affiliations:** ^1^Department of Medical Imaging and Radiological Sciences, College of Medicine, Chang Gung University, Taoyuan, Taiwan; ^2^Department of Medical Imaging and Intervention, Chang Gung Memorial Hospital at Linkou, Taoyuan, Taiwan; ^3^Department of Biomedical Imaging and Radiological Sciences, College of Biomedical Science and Engineering, National Yang Ming Chiao Tung University, Taipei, Taiwan; ^4^iHuman Institute, ShanghaiTech University, Shanghai, China; ^5^Department of Biomedical Imaging and Radiological Science, College of Medicine, China Medical University, Taichung, Taiwan; ^6^Graduate Institute of Biomedical Sciences, College of Medicine, China Medical University, Taichung, Taiwan

**Keywords:** cortical spreading depolarization (CSD), cortical penetrating vessels, cerebral blood volume (CBV), flow velocity, ultrasound dynamic ultrafast Doppler

## Abstract

The dynamic vascular responses during cortical spreading depolarization (CSD) are causally related to pathophysiological consequences in numerous neurovascular conditions, including ischemia, traumatic brain injury, cerebral hemorrhage, and migraine. Monitoring of the hemodynamic responses of cerebral penetrating vessels during CSD is motivated to understand the mechanism of CSD and related neurological disorders. Six SD rats were used, and craniotomy surgery was performed before imaging. CSDs were induced by topical KCl application. Ultrasound dynamic ultrafast Doppler was used to access hemodynamic changes, including cerebral blood volume (CBV) and flow velocity during CSD, and further analyzed those in a single penetrating arteriole or venule. The CSD-induced hemodynamic changes with typical duration and propagation speed were detected by ultrafast Doppler in the cerebral cortex ipsilateral to the induction site. The hemodynamics typically showed triphasic changes, including initial hypoperfusion and prominent hyperperfusion peak, followed by a long-period depression in CBV. Moreover, different hemodynamics between individual penetrating arterioles and venules were proposed by quantification of CBV and flow velocity. The negative correlation between the basal CBV and CSD-induced change was also reported in penetrating vessels. These results indicate specific vascular dynamics of cerebral penetrating vessels and possibly different contributions of penetrating arterioles and venules to the CSD-related pathological vascular consequences. We proposed using ultrasound dynamic ultrafast Doppler imaging to investigate CSD-induced cerebral vascular responses. With this imaging platform, it has the potential to monitor the hemodynamics of cortical penetrating vessels during brain injuries to understand the mechanism of CSD in advance.

## Introduction

Cortical spreading depolarization (CSD) is a slowly propagating (2–6 mm/min) wave of massive neuronal depolarization in the cerebral cortex, causing profound metabolic and hemodynamic responses (Leão, 1944, 1951). CSD is implicated in migraine with aura, and recurrent CSD waves have also been detected in human brain after traumatic brain injury, ischemic and hemorrhagic strokes (Kraio and Nicholson, 1978; Takano et al., 1996; Dreier, 2011). CSD waves propagate from the infarct regions into the surrounding healthy tissue, leading to supply–demand mismatch, neurovascular uncoupling, and hemodynamic disturbance (Fabricius et al., 2006; Shin et al., 2006; Hartings et al., 2017). Considering that CSD is highly associated with brain pathology and poor outcome, understanding the cerebral blood flow (CBF) dynamics during CSD is a critical issue in the management of these neurological disorders (Ayata, 2013; Ayata and Lauritzen, 2015; Kramer et al., 2016).

Different phases of hemodynamic response during CSD have been proposed using various imaging techniques (Ayata and Lauritzen, 2015), including intrinsic optical signal (IOS) imaging (Santos et al., 2014), near-infrared spectroscopy (NIRS) (Seule et al., 2015), laser speckle imaging (LSI) (Woitzik et al., 2013), two-photon laser scanning microscopy (Takano et al., 2007), and photoacoustic imaging (Kirchner et al., 2019). Most studies detected a large transient CBF increase, a peak hyperemia, during CSD under physiological conditions in humans and many animal species, including rats (Dreier et al., 2009). An initial hypoperfusion and a delayed long-lasting oligemia were reported before and after the peak hyperemia, respectively, in certain circumstances (Zhang et al., 1994). Taken together, an initial vasoconstriction followed by vasodilation after the depolarization onset and the delayed vasoconstriction after CSD were, thus, proposed resulting in CBF response during an episode of CSD. Nevertheless, given the limited measurement depth of the traditional optical imaging, the aforementioned dynamic vasomotor response during CSD was mostly derived based on the observation of pial vessels and contiguous cortical arterioles close to the surface of the brains (Brennan et al., 2007). Different segments and compartments of the cerebral vasculature might respond differently to CSD. The penetrating vessels, including penetrating arterioles and venules, are particularly important compartments in bridging the surface and subsurface vascular networks in the cortex (Blinder et al., 2013). Nevertheless, direct measurements from penetrating vessels, as to how cerebral blood volume (CBV) and blood flow velocity change during CSD, are still lacking.

Ultrasound ultrafast Doppler, also termed functional ultrasound (fUS), is an ultrasensitive and quantitative microvascular imaging technique that is able to access hemodynamics of brain and neurovascular coupling, expanding the field of application of ultrasound imaging and providing highly sensitive anatomical and functional mapping of vessels. With high-frequency ultrasound, it can provide high spatiotemporal Doppler imaging (<100 μm, <1 ms) to monitor perfusion change, even arterioles and venules within the whole brain of small animals. Ultrafast Doppler gained sensitivity primarily based on the ultrafast imaging (Tanter and Fink, 2014), which is achieved by the plane wave imaging with reduced transmission time. The imaging frame rate is up to 2k∼10k frames per second (fps). In addition, the spatial resolution and flow sensitivity can be further improved using multiple plane wave coherent imaging to form a high-quality image, and then advanced spatiotemporal clutter filters can be conducted for differentiating low blood flow from stationary tissues as proved by recent animal studies (Macé et al., 2011; Osmanski et al., 2014, 2015; Sieu et al., 2015; Demene et al., 2016; Dizeux et al., 2019). With high-frequency ultrasound, the current technique is ultrasensitive to microvessels of ∼70∼100 μm diameter at the depth up to 1 cm (Macé et al., 2011), which has been verified to conduct functional neuroimaging and access brain functional connectivity in small animals (Macé et al., 2011; Osmanski et al., 2014; Sieu et al., 2015; Demene et al., 2016; Dizeux et al., 2019) or even in the behaving primates (Osmanski et al., 2015). Therefore, ultrafast Doppler is sensitive to the perfusion in the brain, particularly power Doppler indicating relative CBV (Macé et al., 2011, 2013; Brunner et al., 2021) and color Doppler providing flow direction and velocity in the individual small vessels (Brunner et al., 2022a).

The hemodynamic change detected by fUS was first published in 2011 in an epileptic model induced by 4-aminopyridine (4-AP) (Macé et al., 2011). By following this pioneering study, such novel and real-time technique using fUS was further applied in 4D imaging to detect functional and epileptiform events in the rodent brain (Rabut et al., 2019). Lately, Brunner et al. (2022b) showed the hemodynamic change of spreading depolarization in the animal model of ischemic stroke. Moreover, the translational value of fUS was demonstrated in human neonates to measure the flow response of neonatal seizures (Demene et al., 2017).

In the present study, we employed dynamic ultrafast Doppler to quantitatively probe the dynamic vascular responses during the propagation of KCl-induced CSD in the cerebral cortex of rats. In addition to the propagating speed and the peak amplitude of relative blood volume during CSD, we further tracked and analyzed the detailed hemodynamic responses in each phase of the triphasic period during CSD. Most importantly, we investigated the CBV and flow velocity changes in individual penetrating vessels and reported the difference in vascular response during CSD between penetrating arterioles and venules.

## Materials and methods

### Animal preparation

The reporting of animal experiments complies with the ARRIVE guidelines. All experiments were conducted according to the guidelines for the Care and Use of Experimental Animals and approved by the Institutional Animal Care and Use Committee at China Medical University (CMUIACUC-2020-378-1). Six Sprague–Dawley (SD) rats (male, 8–12 weeks old; from BioLASCO Taiwan Co., Ltd., Taipei, Taiwan) were used in this study. All animals were kept in groups of 2–3 with free access to laboratory food and water and maintained in a temperature/humidity-controlled room (21–25°C, with a relative humidity of 60 ± 10%) under a 12:12 light/dark cycle (lights on at 8:00 a.m.).

### Surgery

**FIGURE 1:**
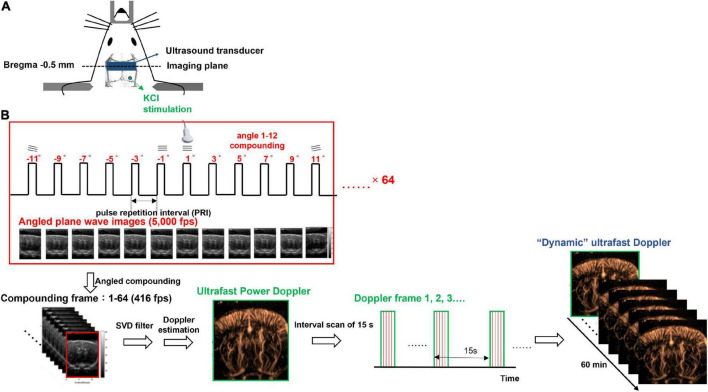
Experimental setup of dynamic ultrafast Doppler. **(A)** Schematic illustration of the cranial window, the position of KCl stimulation, and the imaging plane of the transducer. **(B)** Imaging sequence of dynamic ultrafast Doppler. Twelve angles (from −11° to +11° with a 2° interval) plane wave imaging was conducted at the imaging frame rate of 5k fps to constitute one compounding image (B-mode), and 64 compounding images were acquired for the generation of one Doppler frame (power/color Doppler image). Time-lapse imaging was acquired by sequential recording dynamic ultrafast Doppler frames with an interval of 15 s.

Rats were given 0.04 mg/kg atropine (Sigma-Aldrich, St. Louis, MO, United States), the agent to reduce salivation, subcutaneously 5 min before full anesthesia with combined administrations of 12 mg/kg Zoletil 50 (Virbac Corporation, France) and 10 mg/kg Xylazine (Rompun, Bayer, Germany) intraperitoneally and placed in a stereotaxic apparatus (David Kopf Instruments, Tujunga, CA, United States). After a midline incision to expose the skull, a trapezoidal portion of parietal bone was removed by drilling along the coronal suture and the perimeter, 14 mm parallel with the coronal suture and 8 mm posterior to bregma, to create a cranial window. All drilling processes should be done slowly and gently to avoid dural lesions. Once the parietal bone was removed with forceps, the dura mater should be kept moist with sterile saline. Because of no significant bleeding during craniotomy, all the parietal sinus and central sinus were preserved. Next, the cranial window was enclosed by four plastic pieces fixed on the skull with dental cement and poured the gel as the medium for acoustic coupling between the transducer and the brain. Finally, a circular foramen was created by a high-speed drill (0.9 mm diameter) over the right parietal bone for KCl application. The location of KCl stimulation was at bregma −10 mm on the right hemisphere as shown in Figure 1A. A cotton ball soaked with 2M KCl was placed in the circular foramen and was kept moist during the whole recording to elicit CSD.

### Imaging system setup and dynamic ultrafast Doppler imaging sequence

**FIGURE 2:**
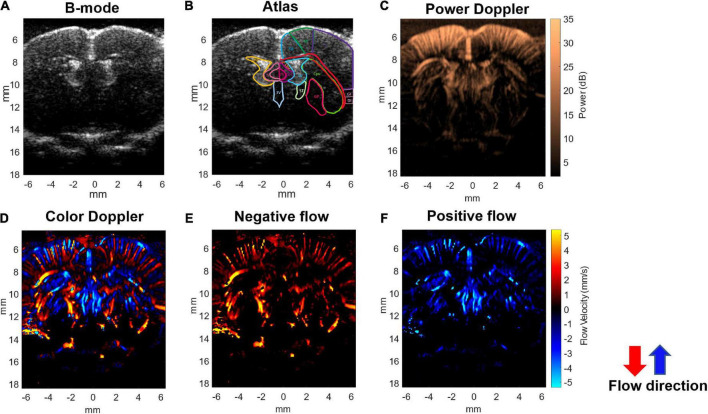
Ultrafast Doppler images of the penetrating vessels in the coronal section of a representative rat brain. **(A)** The representative B-mode image clearly registers the lateral ventricle. **(B)** Corresponding coronal atlas at bregma AP –0.5 mm. **(C)** Ultrafast power Doppler generated from a time series of B-mode images detects the basal blood flow in cerebral cortex. **(D)** The color Doppler image presents flow velocity and directional blood flow toward/outward the ultrasound transducer. **(E)** Negative flow corresponding to blood flow away from the transducer is coded as red. **(F)** Positive flow corresponding to blood flow toward the transducer is coded as blue. In the cerebral cortex, blood in most penetrating arteries and arterioles flows away from the transducer and is coded as red. Blood in most penetrating veins and venules flows toward the transducer and is coded as blue.

An imaging system (Prodigy, S-Sharp, Taiwan) cooperated with a high-frequency transducer was used for ultrafast Doppler acquisition. The transducer was fixed on a 3D linear stage to scan different coronal planes. The plastic chamber filled with ultrasonic gel was placed between the transducer and the brain for acoustic coupling. The location of the imaging plane was 0.5 mm posterior to the bregma as shown in Figure 1A. The lateral ventricle of the brain in ultrasound B-mode image (Figures 2A,B) was used to confirm the imaging location. Once the lateral ventricle was identified, the location of the imaging plane was fixed in coronal orientation over the hind limb primary sensory cortex (S1HL), and the coronal ultrafast Doppler images were sequentially acquired during the whole scan sessions.

The ultrafast Doppler imaging sequence which is a 16.4 MHz and 12 angles (from −11° to +11° with a 2° interval) plane wave imaging at the imaging rate of 5,000 fps was conducted, and 64 compounding images as 768 firings in total were acquired for one Doppler frame. The compounding frame rate is 416 fps to track Doppler signals in the brain. The imaging processing is based on the ultrafast Doppler technique followed by a singular value decomposition (SVD) filter to extract brain flow signals from stationary tissues. The energy distribution of Doppler blood flow can be evaluated by the incoherent correlation matrix of spatial singular vectors obtained by the SVD filter (Baranger et al., 2018). The range of singular values in our study was 12–60 and applied to all animal data. The removal of 11 first singular values corresponding to the stationary tissue signals and four last singular values corresponding to high-frequency electronic noises were filtered out. The use of the filter can effectively differentiate the static signal of tissue and micro-blood flow, and then, Doppler estimation was performed to access the perfusion. The intensity of power Doppler image (Figure 2C) is proportional to the volume of blood in the voxel as an estimator of the relative cerebral blood volume (rCBV) (Macé et al., 2011, 2013; Brunner et al., 2021), and the color Doppler image (Figure 2D) can provide flow velocity to estimate perfusion in the brain.

**FIGURE 3:**
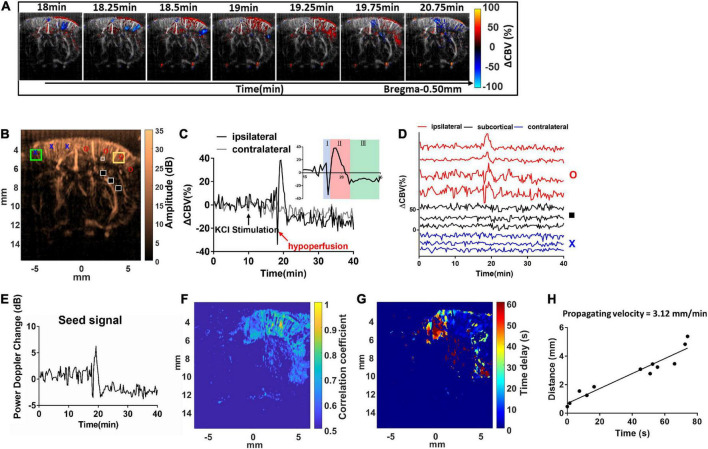
Dynamic ultrafast Doppler images of CBV during a representative episode of CSD. **(A)** Spatiotemporal evolution of CBV changes in rat cerebral cortex in response to CSD acquired by sequence of power Doppler imaging. The color bar was indexed as ΔCBV(%) from –100 to 100%. Positive values representing increase in CBV are coded as red, while negative values representing decrease in CBV are coded as blue. **(B)** The power Doppler image showing the selected ROIs (1 mm × 1 mm) for the wide range analysis in ipsilateral (yellow square) and contralateral cortex (green square), and the ROIs for single-pixel analysis located in ipsilateral (red circle), subcortical (black square), and contralateral (blue cross) regions, and the seed ROI (gray box) for seed-based correlation map. **(C)** Temporal profiles of 1CBV during CSD in the hemisphere ipsilateral (black) and contralateral (gray) to the KCl application site. The black arrow indicates the stimulation of KCl. **(D)** Temporal profile of ΔCBV during CSD from the representative pixels in the ipsilateral cortex to the KCl application site (red), subcortical region (black), and contralateral cortex (blue). **(E)** The representative temporal profile of vascular response extracted from the seed ROI. **(F)** The seed-based correlation map was obtained by subsequently correlating with the temporal profile at each pixel in the whole brain with the seed signal. **(G)** The propagation time delay map was obtained by the phase difference of power Doppler signals between seed point and other regions, and **(H)** the distance–time plot was used to measure the propagating velocity. The slope of the linear regression curve can be measured as the propagating velocity of spreading CSD.

For monitoring dynamic vascular responses, multiple Doppler frames were recorded, and the interval between frames is 15 s. In total, 240 frames were sequentially recorded as the recording period was 60 min to monitor the blood flow change caused by CSD propagation. The data shown in Figure 3C were truncated as the last 40 min to show the flow change caused by CSD. The first 20 min were used to record the resting state basal flow. All of the Doppler frames were processed with the same filtering parameters. Since some power Doppler images randomly show abnormal artifacts due to breath motion, these images were corrected and interpolated by other close and normal Doppler images. The diagram of imaging acquisition is shown in Figure 1B.

### Data analysis

#### Cerebral blood volume estimation

The temporal profiles of CBV changes (ΔCBV_*k*_(%)) within the region of interest (ROI) at time frame *k* were estimated from a time series of power Doppler frames by equation (1).


(1) $$\Delta {\mathrm{CBV}}_{k}\left(\%\right)=100\times \left[\frac{P_{D\,o\,p\,p}\left(k\right)\;-\;\mathrm{avg}.\;(P_{D\,o\,p\,p}\left(1\;:\;15\right))}{\mathrm{avg}.\;\left(P_{D\,o\,p\,p}\left(1\;:\;15\right)\right)}\right]$$

where *P*_*Dopp*_(*k*) is the mean of power Doppler signal over the ROI for Doppler ensemble acquisition of frame *k* and $avg.\left(P_{D\,o\,p\,p}\left(1:15\right)\right)=\frac{\sum_{k=1}^{15}P_{D\,o\,p\,p}\,\left(k\right)}{15}$ which is the mean of power Doppler signal within the ROI of the first 15 frames (from frame 1 to 15) to represent the average power of basal flow before KCl stimulation. The difference between the mean values within the ROI in the sequential Doppler images and that in the basal CBV before the stimulation was calculated, then normalized to the basal CBV baseline, and multiplied by 100. In other words, the temporal profiles were normalized on the basis of the pre-treat level.

Spatial ΔCBV(%) in the brain was estimated, color-coded pixel-by-pixel, and co-registered to the power Doppler images (Figure 3A and Supplementary Video 1). The Δ*CBV*_*k*_(%) can be calculated and tracked in both right (stimulated) side and left (reference) side of the cortex with time. For a wide range analysis, ΔCBV_*k*_(%) was calculated and tracked within the ROI located in the forelimb (S1FL), hind limb (S1HL), or barrel field region (S1BF) of the primary somatosensory cortex in the ipsilateral (yellow square) and contralateral (green square) hemispheres (±3–5 mm from the midline) (Figure 3B). After that, dynamic perfusion changes in the 1 mm × 1 mm ROI (Figure 3C) or even a single pixel (Figure 3D), shown as relative power Doppler change over time. ΔCBV in each phase including the initial hypoperfusion, abrupt elevation, and depression was further quantified. The locations for single-pixel profiles in the ipsilateral (red circle), subcortical (black square), and contralateral (blue cross) regions were labeled in Figure 3B.

**FIGURE 4:**
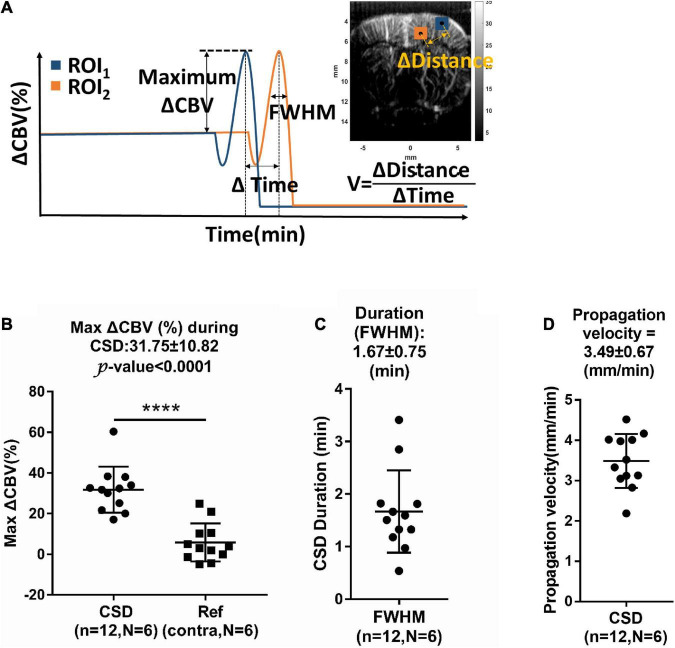
**(A)** Measurement of relative changes in CBV (ΔCBV), full-width half-magnitude (FWHM) of duration, and propagation velocity (V) of hemodynamic changes during CSD. Summarized data of CSD-induced maximum increase in **(B)** ΔCBV, **(C)** FWHM, and **(D)** propagation rate. n: number of CSD; N: number of animals. Data are reported as means ± SD. ****P < 0.0001 between hemispheres.

The maximum ΔCBV was defined as that from the basal flow to the peak of temporal profile. The CSD response duration was defined as the full-width half-magnitude (FWHM) of the peak response (Figure 4A). In addition, the CSD propagation velocity (V) can be estimated by the relation between the cumulative distance and the delay time of arrival on a wide region. Due to the time interval between Doppler frames being 15 s, the traces of hemodynamics were first interpolated by a factor of 10, which corresponds to 1.5-s interval to further calculate the time delay map within the cortex. The reference point was set at the location of seed ROI (40 pixels × 40 pixels), the gray box in Figure 3B, regarding the center to the maximum response caused by CSD stimulation. The correlation coefficient and delay time (phase difference) between Doppler signals at reference point and other pixels within the ipsilateral cortex were calculated with the cross-correlation method. The correlation map (*r* > 0.5) (Figure 3F) can be used as the mask for the time delay map (Figure 3G). The propagation time delay was given by the position of the maximum of the correlation function. Finally, the propagating velocity can be estimated as the slope of the regression line (Figure 3H).

#### Seed-based correlation map of vascular response caused by cortical spreading depolarization

The representative response from a seed ROI (40 pixels × 40 pixels), the gray box in Figure 3B, regarding the center to the maximum response caused by CSD stimulation, was found, and the seed time profile (Figure 3E) was exactly correlated with the temporal profile of CBV variations at each spatial pixel with time in the whole brain. The cross-correlation equation of two discrete time sequences can be expressed by equation (2).


(2) $$R_{x,y}\;\left(k\right)=\frac{1}{Q}\,\sum_{i=0}^{N-1}S_{\mathrm{seed}}\,\left(i\right)\,S_{x,z}\,\left(i\;-\;k\right)$$

where *S*_seed_ is the seed signal, and *S*_*x*,*z*_ is the time profile of each pixel in Figure 3B. *Q* is each discrete time point in the time profiles of *S*_seed_ and *S*_*x*,*z*_. In addition, the temporal profile in Figure 3E is the seed signal used for the calculation of the correlation map shown in Figure 3F. The correlation coefficient map (Figure 3F) shows the responses of blood flow change in the region of brain to CSD.

#### Blood flow velocity image with dynamic ultrafast color Doppler

The color Doppler frames (Figure 2D) were also estimated following the Doppler algorithm after SVD filtering. It was obtained to visualize the 2D distribution of blood flow direction and flow velocity. To compensate for the mean velocity bias with color Doppler estimation caused by the color Doppler algorithm (such as cutoff frequency of high-pass filtering or lower bound of SVD filtering) and the different flow angle to the ultrasound transducer, rather than directly quantify the specific velocity in a single vessel, we calculated the Δflow velocity (%) relative to the basal flow in the same ROI to reduce the bias effect by color Doppler estimation. The flow velocity changes [ΔFlow velocity (%)] were estimated by equation (3).


(3) $$\Delta \;\mathrm{flow}{\mathrm{velocity}}_{k}\left(\%\right)=100\times \left[\frac{V_{D\,o\,p\,p}\left(k\right)\;-\;\mathrm{avg}.\;\left(V_{D\,o\,p\,p}\left(1\;:\;15\right)\right)}{\mathrm{avg}.\;\left(V_{D\,o\,p\,p}\left(1\;:\;15\right)\right)}\right]$$

where *V*_*Dopp*_(*k*) is the mean flow velocity within the ROI in time frame *k* and *avg*.(*V*_*Dopp*_(1 : 15)) is the average of the mean flow velocity within the ROI of the first 15 frames (from frame 1 to 15) to represent the basal flow velocity before KCl stimulation.

#### The different changes of cerebral blood volume and flow velocity in penetration arterioles and venules during cortical spreading depolarization

The penetrating arterioles toward the brain and venules outward to the brain were differentiated based on the direction of blood flow by the color Doppler estimation (Osmanski et al., 2015; Edelman et al., 2021; Brunner et al., 2022a). The ROI (0.1 mm × 0.1 mm) corresponding to the imaging resolution was placed on the descent vessels in the stimulated cortex (within 6 mm from the midline), which were identified from the color Doppler for vessel classification based on their flow direction inward/outward to the transducer. In total, 380 cortical vessels from six animals were identified and represented to access the CBV and flow velocity response contribution of cortical vessels. The maximum ΔCBV(%) and ΔFlow velocity (%) were estimated for comparing the responses to CSD in penetrating arterioles and venules. The correlation of maximum CBV (or flow velocity) and the basal CBV (or basal flow velocity) values in each penetrating vessel was further analyzed.

### Statistical analysis

To compare the maximum ΔCBV response to CSD between the ipsilateral and contralateral cortex, 12 CSD events from six animals were analyzed with the paired *t*-test. To reveal how the penetrating arterioles and venules respond to CSD events, data from 185 venules (138 and 47 venules from the ipsilateral and contralateral cortex, respectively) and 195 arterioles (149 and 49 arterioles from the ipsilateral and contralateral cortex, respectively) during the total 12 CSD episodes were extracted and analyzed. To test whether the collected data were in normal distribution, the Kolmogorov–Smirnov test was performed. Since the data from the venules and arterioles during the CSD event and the ΔCBV data from the venule in the reference hemisphere were not in normal distribution, the ΔCBV and maximal change of flow velocity among different penetrating vessels were compared using the Kruskal–Wallis test, followed by the Mann–Whitney U *post-hoc* test. Data are reported as means ± SD. The significance level was set at *P* < 0.05. Linear correlation analysis was given as R^2^.

## Results

### *In vivo* ultrafast Doppler imaging of the rat brain

Using the ultrafast Doppler imaging system, we obtained the general brain structure with B-mode (Figure 2A), the intensity of power Doppler image proportional to the rCBV (Figure 2C), and color Doppler corresponding to the direction and velocity of blood flow (Figure 2D), respectively. The imaging plane was at −0.5 mm posterior to bregma and was identified according to the coronal section images of the lateral ventricle obtained from B-mode in all experiments (Figure 2B). The power Doppler imaging clearly demonstrated cortical vasculature in the region, including the primary motor cortex and partially the sensory cortex. rCBV in the cortex within ±5 mm lateral to the midline was included in further analysis. The blood flow imaging can reach more than 1.5 cm at least in the brain covering the whole depth of the brain. In the color Doppler imaging, the negative flow coded as red referred to the blood flow streaming outward the transducer (Figure 2E), and the positive flow coded as blue color referred to the blood flow streaming toward the transducer (Figure 2F). In the brain vascular anatomy, the penetrating arteries and arterioles specifically dive radically into the brain parenchyma which flows away to the transducer, ascending venules that drain blood from microcirculatory beds and return to the superficial cortex flow to the transducer. Therefore, penetrating arteries (red) and veins (blue) in the region of cortex can be further differentiated according to the flow directions denoted by the color Doppler imaging (Figures 2D–F). This arteriovenular differentiation based on color Doppler has been reported in previous Doppler works (van Raaij et al., 2012; Macé et al., 2013; Brunner et al., 2022a). The dimensions of penetrating arterioles and venules in our Doppler images were 119 ± 12 and 137 ± 17 mm of arterioles and venules, respectively (Supplementary Figure 1).

### The cortical spreading depolarization propagation wave is indexed by dynamic changes in cerebral blood volume

The hemodynamic changes that accompany the propagation of CSD can be clearly detected with dynamic ultrafast Doppler imaging (Figure 3A and Supplementary Video 1). The color-coded index in Figure 3A and Supplementary Video 1 shows the ΔCBV(%) compared to the basal flow pixel-by-pixel. The red indicated hyperperfusion, and the blue indicated hypoperfusion, respectively. By analyzing the Doppler signals from the specific ROI of 1 mm × 1 mm (Figure 3C), even at each pixel through time (Figure 3D), we observed that KCl stimulation can trigger CBV changes in bi- or triphasic manner. From our results, we observed that KCl stimulation can trigger CBV changes in triphasic manner in most cases, including an initial hypoperfusion caused by transient vasoconstriction (phase I), followed by an apparent increase (phase II) with remarkable vasodilation of cerebral vessels, and lastly, an overshoot (phase III) with long-lasting vasoconstriction as shown in subfigure in Figure 3C. The ROIs for ΔCBV_*k*_(%) at each phase of the CSD event were labeled, and the trends obtained from all animals are shown in Supplementary Figure 2.

In the coronal section, these CBV changes progress as a wave and are initiated from the superficial layer of the right cortex after the stimulation and then propagated outward across the whole depth of the right cortex in a time-wise manner indicated by multiple time-lapse frames of Doppler images (Figure 3A and Supplementary Video 1).

The KCl-triggered vascular dynamics do not propagate across the midline to invade the contralateral hemisphere or penetrate adjacent subcortical regions. CSD-induced vascular dynamics are also constrained to the same side of the cortex with KCl stimulation (Figures 3C,D). Figure 3E shows the power Doppler difference of time series individual frames *P*_*Dopp*_(*k*) compared to the basal flow *avg*.(*P*_*Dopp*_(1:15)) within the seed ROI, which is the extent of the time profile used for the calculation of the correlation map. Then, we further analyzed the seed-based correlation map, demonstrating that the propagation wave of the CBV dynamics which is corresponding to high correlation coefficient shown in Figure 3F was confined within the ipsilateral cortex to the stimulation site.

The maximum ΔCBV during CSD in the stimulated hemisphere was 31.75 ± 10.82% to the basal level within the same ROI and has a significant difference compared to the contralateral hemisphere. The average CSD duration was 3.09 ± 1.31 min and 1.67 ± 0.75 min in the whole period and FWHM of peak, respectively, as shown in Figure 4C. The mean propagation speed is 3.49 ± 0.67 mm/min by measuring the slope of regression line between the distance to the reference point and the delay time (Figure 4D).

### The cerebral blood volume dynamics to cortical spreading depolarization

Significant biphasic or triphasic changes were observed upon the KCl stimulation compared with the basal flow. The first phase of CSD-induced CBV dynamics is characterized as initial hypoperfusion (−9.51 ± 8.54% in CBV), which was clearly observed in 11 out of 12 CSD episodes from six animals (red arrows in Figure 3C and Supplementary Figure 2). The secondary phase is the abrupt elevation of the CBV by 31.75 ± 10.82% relative to the basal value. Finally, the average CBV level is about −7.96 ± 5.78% lower than the basal level during this phase. The results were consistent with the CBV changes of −5∼−30%, 30∼250%, and −10∼−40% reported by the previous studies (Ayata and Lauritzen, 2015).

After these dramatic CBV changes, the basal level was stable unless the coming of the next CSD wave. From the 1-h experimental session, we did not observe the flow back to the original basal level after the KCl application. This delayed oligemia may keep for 1–2 h or the following hyperemia triggered by the latter CSD, and it may keep even longer to 3 days reported by the previous work (Otori et al., 2003).

### The different changes of cerebral blood volume and flow velocity in penetration arterioles and venules during cortical spreading depolarization

**FIGURE 5:**
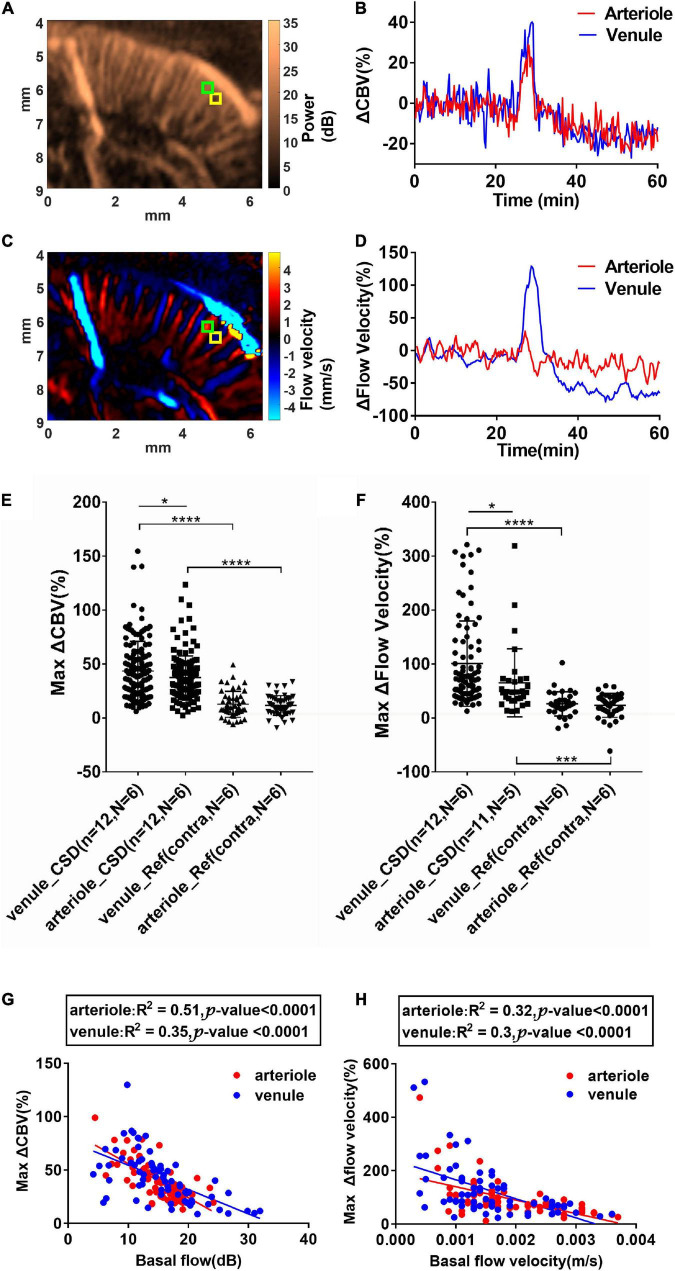
Cortical spreading depolarization-induced CBV and flow velocity changes in single penetrating vessels. **(A)** Power Doppler and **(C)** color Doppler images showing CBF in penetrating arterioles and venules. Blood flows toward the transducer and away from the transducer are labeled as blue and red, respectively. **(B)** ΔCBV and **(D)** Δflow velocity changes in the single penetrating arteriole and venule as indicated in ROIs in **(A)** and **(C)** (arteriole: Green ROI and venule: Yellow ROI). Summarized data of maximum ΔCBV and maximum Δflow velocity in single penetrating vessels during CSD. **(E)** Maximum ΔCBV in single arterioles and venules in the ipsilateral and contralateral side of KCl stimulation. **(F)** Maximum Δflow velocity increase in single arterioles and venules in the ipsilateral and contralateral sides of KCl stimulation. Data are reported as means ± SD. Statistical significance is determined by two-tailed Student’s *t*-test and defined as *p < 0.05; ***p < 0.001; ****p < 0.0001; n.s., no significance *P* > 0.05. **(G)** Correlation of maximum CBV increases and the basal CBV values in single penetrating vessels. **(H)** Correlation of maximum flow velocity increases and the basal flow velocity values in single penetrating vessels. Blood flows toward the transducer (penetrating venules) and away from the transducer (penetrating arterioles) are labeled as blue and red, respectively. The correlation was analyzed using Pearson’s correlation coefficient analysis.

In rodents, the penetrating venules drain blood from microcirculatory beds and return to the superficial cortex, whereas the penetrating arterioles specifically dive radically into the brain parenchyma (Blinder et al., 2013). Based on color Doppler images (Figures 2D–F), we measured ROIs located within a single arteriole or venule to estimate the relative change of CBV (ΔCBV) (Figures 5A,B) and relative flow velocity of single vessels (Figures 5C,D) in a representative animal. The size of ROI used to reflect the flow velocity response is 0.1 mm × 0.1 mm. Data from 185 venules (138 from CSD hemisphere and 47 from reference hemisphere) and 195 arterioles (146 from CSD hemisphere and 49 from reference hemisphere) during 12 CSD episodes in six animals were analyzed. The selected ROIs and analysis for each animal were shown in Supplementary Figure 3. The penetrating venules and arterioles exhibit ΔCBV in response to CSD (45.50 ± 36.39% and 37.32 ± 20.11% increase in venules and arterioles, respectively, compared to the baseline, *P* < 0.05, Figure 5E). Furthermore, the maximal change of flow velocity is 101 ± 78.74% in venule whereas only 65.28 ± 62.25% in the arterioles (*P* < 0.05, Figure 5F). The response difference in arteriole and venule was more prominent in flow velocity change than in CBV. The representative ΔCBV and ΔFlow velocity data in arteriole and venule are shown in Figures 5B,D to investigate the same finding in the statistical analysis. In conclusion, it is indicating a wider dynamic range of CBV and flow velocity increase in the venules. The analysis from all the animals about the correlation of max ΔCBV (middle column) and max Δflow velocity (right column) vs. basal flow is shown in Supplementary Figure 3. The changes in CBV value and flow velocity were negatively correlated with the basal CBV in both arteries and veins. More interestingly, the changes in CBV value were negatively correlated (*R*^2^ = 0.51, *P* < 0.0001 for arterioles; *R*^2^ = 0.35, *P* < 0.0001 for venule) with the basal CBV in both arteries and veins, indicating that the small vessel has the wider dynamic ranges (Figure 5G). The velocity changes have no obvious relation with the basal flow velocity (Figure 5H).

## Discussion

In this study, we proposed using ultrasound dynamic ultrafast Doppler technique to investigate CSD-induced propagating cerebral vascular responses of the brain in anesthetized rats *in vivo*. The hemodynamics of cortical penetrating vessels showed triphasic changes, including initial hypoperfusion, prominent hyperperfusion peak, followed by a long-period depression in CBV to the basal level. Moreover, further quantification of CBV and flow velocity revealed different hemodynamics between individual penetrating arterioles and venules. These results indicate specific vascular dynamics of cerebral penetrating vessels and different contributions of penetrating arterioles and venules to the CSD-related pathological vascular consequences. With this imaging technique, it has the potential to monitor the hemodynamics of brain during brain injuries to understand the mechanism of CSD in advance.

Although the cerebral hemodynamic changes of CSD have been studied using various techniques, including traditional laser Doppler flowmetry, LSI, NIRS, IOS imaging, and two-photon microscopy (Takano et al., 2007; Woitzik et al., 2013; Santos et al., 2014; Seule et al., 2015; Kirchner et al., 2019), the imaging depth of these methods is limited to only depict the signal from the pial vessels or vessels close to the surface of the brain (Dirnagl et al., 1989). In addition, the optical property of absorption and scattering that both contributed from the related chromophores (oxyhemoglobin, deoxyhemoglobin, cytochromes, FAD, NADH, etc.) has to be differentiated carefully to accurately delineate the hemodynamic change using optical approaches (Yin et al., 2013). While two-photon fluorescent imaging is able to provide high-resolution images of vascular network and accurate measurements of vessel diameter changes, it is limited by the visualization of depth limited to hundreds of micrometers and sufficient temporal information of the whole-brain mapping. Compared with these conventional techniques, functional ultrafast Doppler imaging is a quantitative flow mapping to obtain large-field images with high spatiotemporal resolution. With high-frequency ultrasound of 16.4 MHz, the microflow in the whole-brain scale (with an imaging depth of more than 1.5 cm) can be observed with highly sensitive ultrafast Doppler imaging. Not only the penetrating vessels at the cortical level, but the territory of posterior striatal artery and the lateral choroidal vein around the striatum were identified in our presentative images (Figures 2C–F). As it is acknowledged that propagation of CSD was only confined to the ipsilateral cortex (Ayata, 2013; Kao et al., 2014; Ayata and Lauritzen, 2015), we focused on our quantitative analysis in the brain parenchyma and the penetrating vessels in the cortex for the current study.

While, in the same animal, the basic CBV level was stable throughout the whole experiment with fluctuation (1–2%) in the contralateral cortex (Figure 3C), significant changes in CBV were delineated in the ipsilateral cortex in response to CSD, which is not able to penetrate the subcortical tissues. These results are consistent with previous studies that the electrical signals of CSD do not normally cross the brain regions without gray matter connections (Eikermann-Haerter et al., 2011). By using ultrafast Doppler imaging, the triphasic change of CBV composed of (1) an initial decrease (−9.51 ± 8.54%, phase I) due to transient vasoconstriction, (2) followed by an apparent increase (31.75 ± 10.82%, phase II) with remarkable vasodilation of cerebral vessels, and (3), lastly, an overshoot (−7.96 ± 5.78%, phase III) with prolonged vasoconstriction was reported responding to CSD. The transient oligemia (phase I) was detected with high spatiotemporal resolution ultrafast Doppler; only 8.3% (1/12) episodes of CSD showed biphasic data with the loss of phase I in our experiment very likely due to the individual animal condition. Both triphasic and biphasic waveforms were reported as typical hemodynamic changes during CSD (Ayata and Lauritzen, 2015). Our CBV increment (31.75 ± 10.82%) during hyperemia, the most prominent alteration in CSD episodes proposed by different approaches, is in line with 37% enhancement measured by the reflectance of IOS (Chang et al., 2010). The propagation velocity (3.49 ± 0.67 mm/min) of the CBV signal is consistent with standard CSD measured with optical or electrical signals in both previous *in vivo* and *in vitro* studies (Hadjikhani et al., 2001; Zhou et al., 2013).

In contrast to the previous findings showing the flow changes in the pial vessels based on various optical imaging tools, we took the advantage of fUS and reported the hemodynamic change along the cortical penetrating vessels during CSDs. The calculated range of flow velocity in cortical arterioles and venules was 1∼9.7 mm/s and 1∼9.4 mm/s, respectively, in our data. Due to the bias in velocity measurement by using ultrasound color Doppler, our results might be underestimated compared with the flow velocity ranging from 2 to 12 mm/s for penetrating venules and up to 18 mm/s for penetrating arterioles detected by ultrasound ultrafast Doppler spectrum (Brunner et al., 2022a). For ultrasound color Doppler imaging, the estimated mean flow velocity might be dependent on the applied velocity estimation with autocorrelation method, cutoff frequency of high-pass filtering (or SVD filtering), which is for removing stationary tissues, and the angle of flow direction to the ultrasound transducer. In addition, the lowest measured velocity was limited by the cutoff frequency (lower bound of SVD filter), and the highest measured level was limited by the PRF (416 fps for compound images). To reduce the intrinsic velocity estimation bias in our study, rather than absolute value, we calculated the Δflow velocity (%) to denote the hemodynamic change during CSD by normalizing it to the corresponding basal flow in the same vessel. Taking advantage of our technique, the different changes in the CBV and flow velocity between penetrating venules and arterioles during CSD were observed. Our results demonstrate that different patterns of CBV and flow velocity change between penetrating arterioles and venules during CSD (Figures 5B,D). The greater elevation of venous flow velocity compared with that of the arterioles (Figure 5F) is consistent with a previous study showing that the pial venules contribute more than that of pial arterioles during CSD-induced CBF increase (Nielsen et al., 2000).

In addition to the difference between the arterioles and venules, the CBV response to CSD may be associated with the diameter of vessels. From our observation in Figure 5G, a significant negative correlation between the basal CBV levels and CBV increase was observed regardless of the penetrating arteries or veins. As the basal CBV levels, in general, are proportional to the diameter of the vessel (Kobari et al., 1984), our data suggest that CBV in smaller vessels regardless of the arterioles and venules increased to a greater extent in percentage during CSD. In the early studies, Leao reported that, during CSD, the diameter of pial arterioles increased by 50–100% of the basal level (Leo, 1944). Later studies found that CSD-induced dilation appeared to be more prominent in the smaller caliber arterioles than the larger ones (Shibata et al., 1990). The diameter change appeared to be more prominent in smaller caliber arterioles than larger ones which were also reported in the previous studies (Ayata and Lauritzen, 2015). Taken together, we suggest that the small vessels in the cortex, regardless of the pial or penetrating one, may respond to CSD even more vigorously than the larger vessels. Of note, the smallest vessel size delineated by ultrafast Doppler imaging is limited by the spatial resolution of 118.11 μm in lateral measured by our B-mode image. The dimensions of penetrating arterioles and venules in our Doppler images were 119 ± 12 and 137 ± 17 μm of arterioles and venules, respectively (Supplementary Figure 1), which were larger than the proposed size of most penetrating arterioles (10∼50 μm) and venules (10∼100 μm) (Macé et al., 2013; Brunner et al., 2022a). However, based on the imaging principle, as long as the blood flow sensitivity of ultrafast Doppler imaging is high enough, the flows in the vessel with the diameter less than the spatial resolution can still be measurable by the US. Therefore, the mean velocity measured across the dimension of single cortical vessels can still be reported using ultrafast Doppler (Brunner et al., 2022a).

Based on the propagation speed of CSD (about 2–6 mm/min) (Leão, 1944, 1951), the setting of acquisition interval (15 s) with a total period of 1 h should be able to capitulate the dynamic change during the CSD event. The fUS frame rate can be further improved to increase the temporal resolution and also can reach the real-time 3D fUS in the future. The imaging system and probe we used in the current study were 1D linear array to acquire 2D coronal/or sagittal plane images. While we can acquire multiplane images sequentially by using high-speed mechanical scanning and reconstructing the corresponding vasculature maps in the whole depth of the brain, it was possible to synchronously monitor the hemodynamic change to CSD in 3D mapping. The following work by using the 2D array probe will be also proposed to observe the real-time 3D flow changes in the whole-brain scale, including the pial matters on top of the brain surface.

## Data availability statement

The raw data supporting the conclusions of this article will be made available by the authors, without undue reservation.

## Ethics statement

This animal study was reviewed and approved by the Institutional Animal Care and Use Committee at China Medical University.

## Author contributions

B-YH, Y-CK, NZ, and D-CW: conception and design of the study and review and editing of the manuscript. Y-PL and B-YH: imaging acquisition. Y-YM: animal preparation. Y-PL: data management and statistical analysis. B-YH: writing the first draft of the manuscript. S-YC: helped to supervise the project. All authors contributed to the article and approved the submitted version.

## Supplementary material

Supplementary Figure 1 Shows **(A)** the downward blood flow mapping and **(B)** the upward blood flow mapping. The diameter of representative cortical penetrating arterioles (#1, #2, and #3) and penetrating venules (#4, #5, and #6) was further analyzed as 119±12 μm (115, 109, and 133 μm) for arterioles and 137±17 μm (151, 141, and 118 μm) for venules shown in the color Doppler images.

Supplementary Figure 2 The trends from the ROIs in the ipsilateral (yellow box) and contralateral (green box) on all animals. The black arrow indicates the timing of KCl stimulation, and the red arrows indicate the hypoperfusion in the first phase of hemodynamic change caused by CSD. Twelve episodes of CSD-induced hemodynamic changes were observed in six animals.

Supplementary Figure 3 The left column shows the selected ROIs within a single penetrating arterioles marked as red circle and venules marked as blue cross indicated on the ultrafast Doppler map. The middle column represents the correlation between maximum CBV increases and the basal CBV values in single penetrating vessels. The right column represents the correlation between maximum flow velocity increases and the basal flow velocity values in single penetrating vessels. The data are from six animals.

Supplementary Video 1 Shows the representative video of the hemodynamic changes in the whole brain during CSD. The left video (grayscale) shows the time series of power Doppler; the right video shows the time series of ΔCBV (%) coded with red (positive as hyperperfusion) and blue (negative as hypoperfusion), respectively, and co-registered to the power Doppler shown in grayscale.
