# Supplementary figures and images for: Vascular responses of penetrating vessels during cortical spreading depolarization with ultrasound dynamic ultrafast Doppler imaging
Source: Front Neurosci. 2022 Nov 16;16:1015843. doi: 10.3389/fnins.2022.1015843 (PMC9714680; doi:10.3389/fnins.2022.1015843)

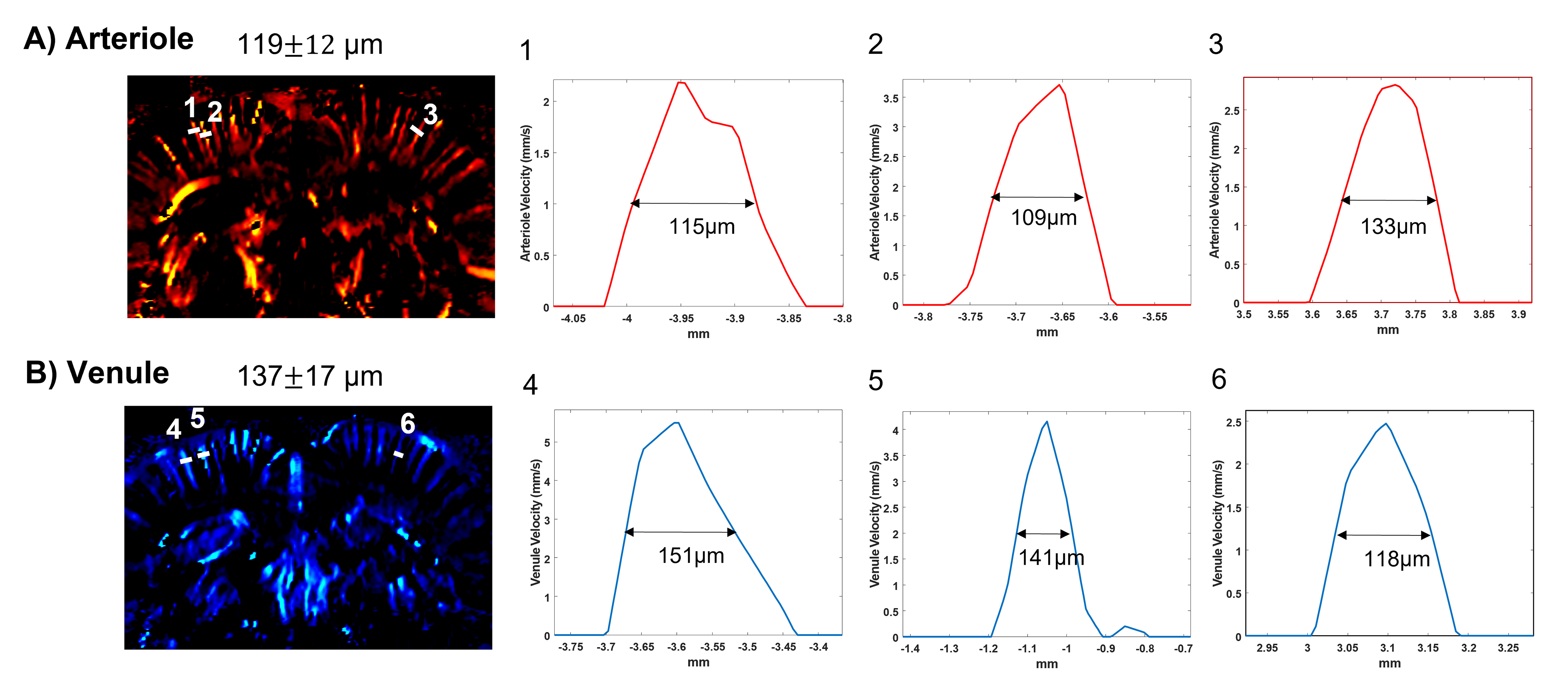

Supplement: Supplementary Figure 1 — Shows (A) the downward blood flow mapping and (B) the upward blood flow mapping. The diameter of representative cortical penetrating arterioles (#1, #2, and #3) and penetrating venules (#4, #5, and #6) was further analyzed as 119±12 μm (115, 109, and 133 μm) for arterioles and 137±17 μm (151, 141, and 118 μm) for venules shown in the color Doppler images. [file Image_1.TIF]

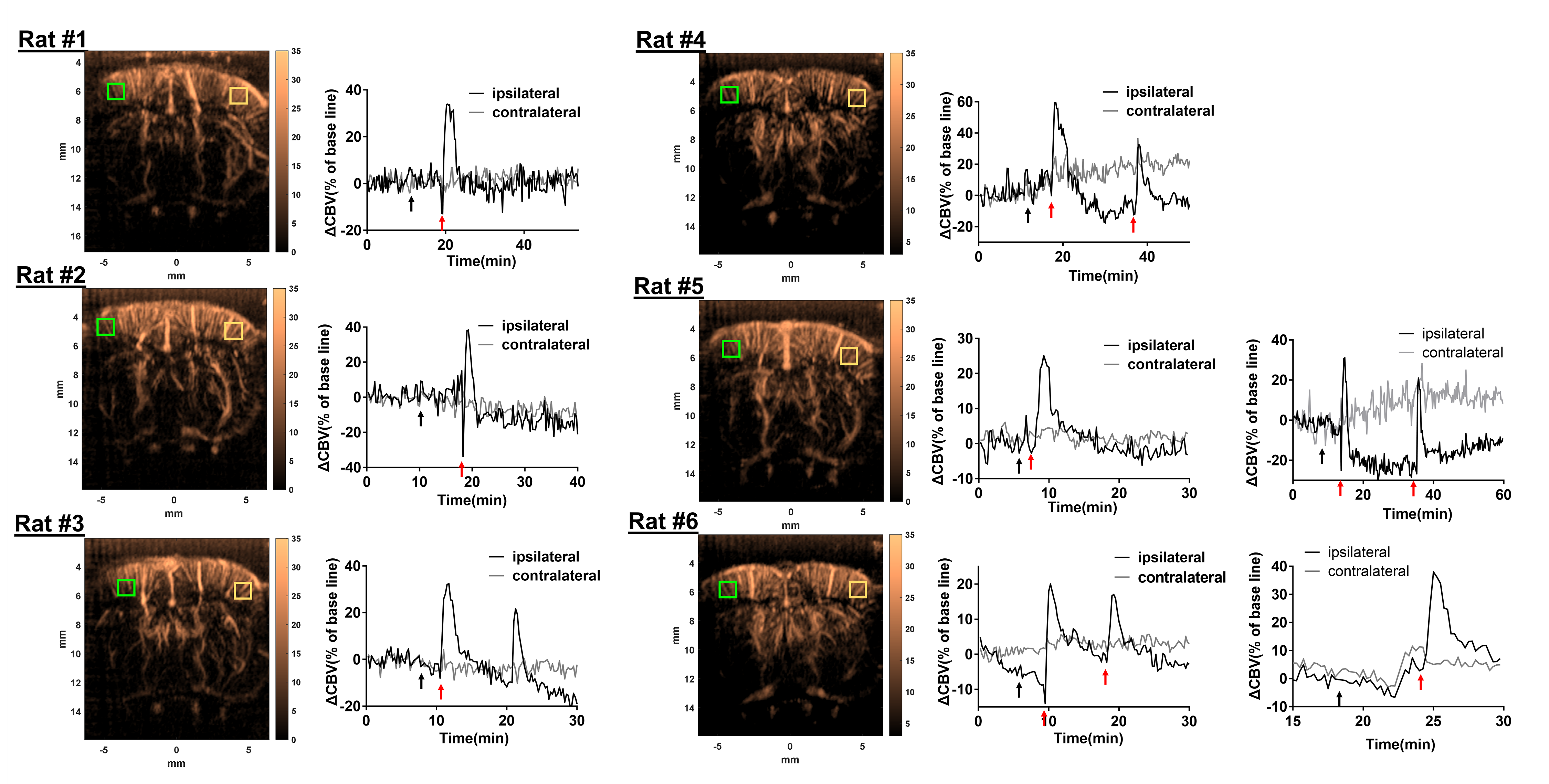

Supplement: Supplementary Figure 2 — The trends from the ROIs in the ipsilateral (yellow box) and contralateral (green box) on all animals. The black arrow indicates the timing of KCl stimulation, and the red arrows indicate the hypoperfusion in the first phase of hemodynamic change caused by CSD. Twelve episodes of CSD-induced hemodynamic changes were observed in six animals. [file Image_2.TIF]

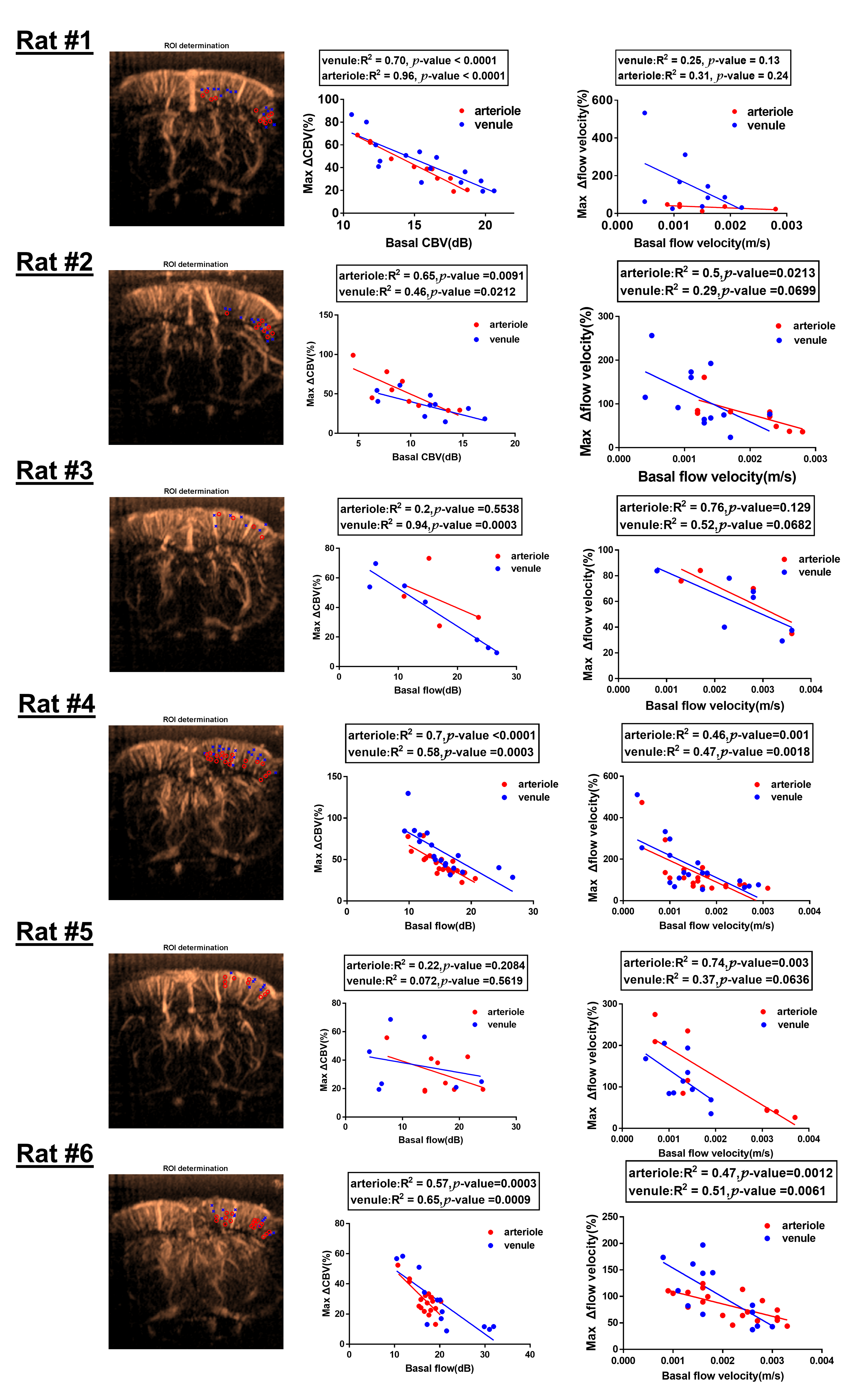

Supplement: Supplementary Figure 3 — The left column shows the selected ROIs within a single penetrating arterioles marked as red circle and venules marked as blue cross indicated on the ultrafast Doppler map. The middle column represents the correlation between maximum CBV increases and the basal CBV values in single penetrating vessels. The right column represents the correlation between maximum flow velocity increases and the basal flow velocity values in single penetrating vessels. The data are from six animals. [file Image_3.TIF]
